# Coevolutionary Analysis Implicates Toll-Like Receptor 9 in Papillomavirus Restriction

**DOI:** 10.1128/mbio.00054-22

**Published:** 2022-03-21

**Authors:** Kelly King, Brendan B. Larsen, Sophie Gryseels, Cécile Richet, Simona Kraberger, Robert Jackson, Michael Worobey, Joseph S. Harrison, Arvind Varsani, Koenraad Van Doorslaer

**Affiliations:** a School of Animal and Comparative Biomedical Sciences, University of Arizona, Tucson, Arizona, USA; b Department of Ecology and Evolutionary Biology, University of Arizona, Tucson, Arizona, USA; c Department of Microbiology, Immunology and Transplantation, Rega Institute, KU Leuven, Leuven, Belgium; d Department of Biology, University of Antwerp, Antwerp, Belgium; e The Biodesign Center for Fundamental and Applied Microbiomics, Center for Evolution and Medicine, School of Life Sciences, Arizona State University, Tempe, Arizona, USA; f The BIO5 Institute, University of Arizona, Tucson, Arizona, USA; g Department of Chemistry, University of the Pacific, Stockton, California, USA; h Structural Biology Research Unit, Department of Integrative Biomedical Sciences, University of Cape Town, Observatory, Cape Town, South Africa; i Department of Immunobiology, University of Arizona Tucson, Arizona, USA; j Cancer Biology Graduate Interdisciplinary Program, University of Arizona Tucson, Arizona, USA; k UA Cancer Center, University of Arizona Tucson, Arizona, USA; Tufts University School of Medicine; University of Pittsburgh

**Keywords:** Mexican free-tailed bat, *Papillomaviridae*, TLR9, evolutionary biology, innate immunity, papillomavirus, speciation

## Abstract

Upon infection, DNA viruses can be sensed by pattern recognition receptors (PRRs), leading to the activation of type I and III interferons to block infection. Therefore, viruses must inhibit these signaling pathways, avoid being detected, or both. Papillomavirus virions are trafficked from early endosomes to the Golgi apparatus and wait for the onset of mitosis to complete nuclear entry. This unique subcellular trafficking strategy avoids detection by cytoplasmic PRRs, a property that may contribute to the establishment of infection. However, as the capsid uncoats within acidic endosomal compartments, the viral DNA may be exposed to detection by Toll-like receptor 9 (TLR9). In this study, we characterized two new papillomaviruses from bats and used molecular archeology to demonstrate that their genomes altered their nucleotide compositions to avoid detection by TLR9, providing evidence that TLR9 acts as a PRR during papillomavirus infection. Furthermore, we showed that TLR9, like other components of the innate immune system, is under evolutionary selection in bats, providing the first direct evidence for coevolution between papillomaviruses and their hosts. Finally, we demonstrated that the cancer-associated human papillomaviruses show a reduction in CpG dinucleotides within a TLR9 recognition complex.

## INTRODUCTION

Papillomaviruses (PVs) are circular double-stranded DNA (dsDNA) viruses found in many hosts, including mammals, reptiles, birds, and fish (1–4). For humans, roughly 400 genetically diverse papillomavirus types have been described. While a subset of these viruses is associated with (malignant) tumors, most viral types do not cause disease in immunocompetent hosts. As with humans, hosts that have been thoroughly sampled are infected with an extensive repertoire of highly diverse yet species-specific viruses. Coevolution of virus and host alone is insufficient to explain the phylogeny of viruses in the family *Papillomaviridae*. For example, papillomaviruses infecting humans do not form a monophyletic group; within the papillomavirus family member, the phylogenetic tree suggests multiple evolutionary mechanisms associated with host cellular interactions and immune evasion as important factors throughout viral genome evolution (1, 5).

Furthermore, host or tissue tropism is likely a significant determinant of host-pathogen interactions (6–8). Therefore, genomic analyses that consider essential mechanisms of the viral life cycle and evolutionary pressures related to host-parasite interactions may provide a novel perspective into papillomavirus genome evolution. A broader description of animal viruses will continue to inform these efforts.

A successful infection requires that the papillomavirus DNA is delivered to the host cell nucleus. Papillomaviruses access mitotically active basal cells through lesions in the stratified epithelia of cutaneous or mucosal tissues. Following binding to cellular receptors and priming by kallikrein-8 and furin cleavage, the virus is endocytosed (9–15). The viral DNA is transported to the Golgi before the mitosis-dependent nuclear accumulation of L2 and viral DNA near promyelocytic leukemia (PML) bodies (14, 16–23).

Host cells detect a variety of viral pathogen-associated molecular patterns (PAMPs) by pattern recognition receptors (PRRs), resulting in induction of interferon (IFN) and a potent antiviral response (24). The concerted actions of PRR signaling, specific viral-restriction factors, and viral evasion strategies determine the eventual outcome of viral infection (25).

The papillomaviral structural proteins (L1 and L2) have no known enzymatic activity to directly counteract antiviral responses (26, 27). Instead, we demonstrated that papillomaviruses evolved an elaborate trafficking mechanism to evade PRR sensing pathways within the cytosol (28–30). Furthermore, millions of years of virus-host cospeciation left historical evidence of immune evasion events in these viruses’ genomes (31). For example, APOBEC3 has been demonstrated to restrict infection with human papillomavirus (HPV) (32, 33). We previously demonstrated that alphapapillomaviruses are significantly depleted of TpC dinucleotides, the target for APOBEC3-mediated mutagenesis. This TpC depletion evolved as a mechanism to evade APOBEC3-mediated mutagenesis. Specifically, this depletion of the TpC content is more pronounced in mucosal alphapapillomaviruses and is correlated with significantly higher expression levels of APOBEC3 in mucosal tissues (33). These findings illustrate that host antiviral activity plays a critical role in regulating papillomavirus evolution and that we can use “molecular archeology” to identify these events.

Toll-like receptors (TLR) survey the extracellular and endosomal compartments and represent the first defense line against foreign invaders. TLR2 and TLR4 recognize viral glycoproteins (34–40), TLR3 recognizes double-stranded RNA (41–46), and TLR7 and TLR8 recognize viral single-stranded RNA (47–53). The endosomal TLR9 detects unmethylated CpG motifs found in dsDNA (viral) genomes (25, 40, 54, 55).

As described above, HPV particles are endocytosed (20, 29), and viral DNA could be recognized by endosomal TLR9, resulting in a downstream inflammatory immune response. We hypothesize that TLR9 may detect papillomavirus dsDNA leading to CpG depletion, similar to what we observed for APOBEC3 target motifs. Indeed, papillomavirus genomes have reduced CpG content (33, 56). This overall dinucleotide depletion confounds the ability to demonstrate the cause of this depletion.

Bats serve as reservoirs for many viruses and have served as the source of well-documented cross-species transmission of pathogens responsible for myriad epidemics, the most notable being severe acute respiratory syndrome coronavirus 1, Middle East respiratory syndrome coronavirus, Ebola virus, Marburg virus, and, most recently, severe acute respiratory syndrome coronavirus 2 (57–59). It has been proposed that bats avoid immunopathological outcomes by not fully clearing a viral infection (60). Indeed, bats have been reported to exhibit a “dampened” immune response to viral infections (57, 61–64). The complex suppression of immune response pathways is variable between several bat species (order Chiroptera) (63, 65–67). Importantly, it was demonstrated that residues involved in the ligand-binding region of the bat TLR9 protein are evolving under positive selection (65, 66). This evolutionary selection of TLR9 has been proposed to contribute to the high tolerance for viral infections observed in bats (57, 67). Notably, the coevolution theory suggests that viruses need to counter these changes in TLR9, and we should be able to detect these host-parasite interactions (33, 68).

To address this issue, we determined the genomes of two novel bat papillomaviruses from Tadarida brasiliensis (TbraPV2 and TbraPV3). Taxonomically, bats are classified into two suborders, Yinpterochiroptera and Yangochiroptera. By comparing the genomes of papillomaviruses associated with bats from either suborder, we demonstrate that TLR9 target motifs are significantly depleted and impact papillomavirus evolution. Furthermore, we extend existing data showing that Yangochiroptera TLR9 evolves under diversifying selection. These data argue that papillomavirus genomes evolve in response to a host change. This is the first direct evidence of PVs evolving in response to host evolutionary changes, thus providing direct evidence for coevolution. Also, these data argue that TLR9 is a restriction factor for papillomavirus infection.

## RESULTS

### Sampling, sample processing, and viral metagenomics.

We captured bats in mist nets set over water sources, extracted them from the nets, and put them in brown paper bags. Feces were collected and processed for high-throughput DNA sequencing as described previously (69). We identified two circular contigs (circular based on terminal redundancy) that had similarities to papillomavirus sequences. We mapped the raw reads to the assembled genomes using BBmap (70) to determine the read depth. We had a 22× to 25× coverage depth across the genome, with 1,200 to 1,300 mapped reads for both genomes.

### New bat-associated papillomaviruses cluster with previously identified chiropteran viruses.

**FIG 1:**
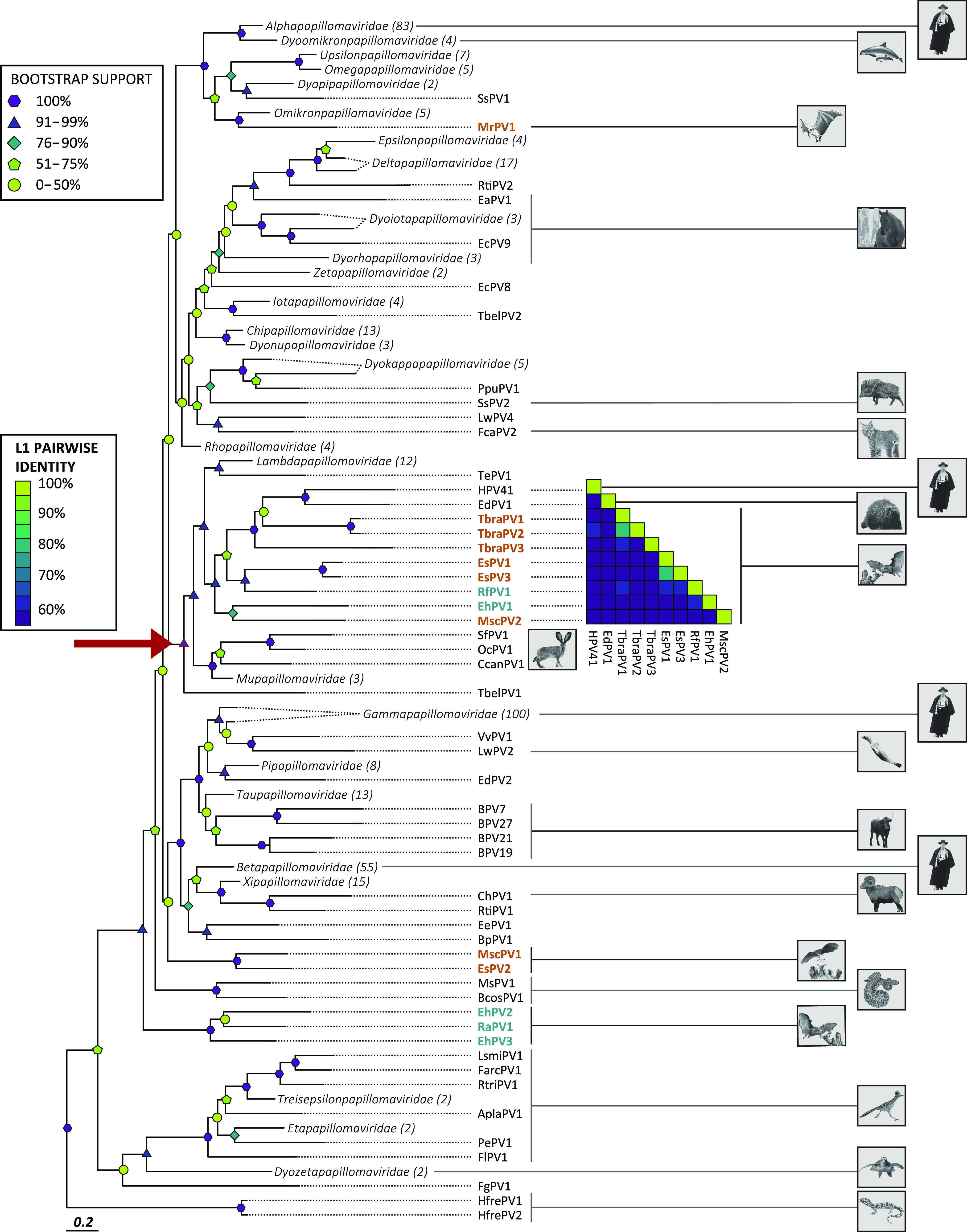
Evolutionary relationship of novel bat papillomaviruses. Shown is a maximum-likelihood phylogenetic tree inferred using concatenated E1, E2, and L1 protein sequences. Papillomaviruses associated with Chiroptera are highlighted: Yangochiroptera (orange) and Yinpterochiroptera (green). Papillomavirus genera are collapsed (the number of types within each genus is indicated in parentheses). Bootstrap-generated branch support values are given using symbols and color gradients. Host species are shown using Sonoran Desert-dwelling animals. The red arrow indicates the subtree used for further analyses as described throughout the paper. Inset plots the percentage pairwise identities across the L1 nucleotide sequences.

We determined the genomes of two novel circular dsDNA viruses using a metagenomics approach. The open reading frames (ORFs) of these putative new viruses were identified using PuMA (150). This analysis identified the typical papillomavirus open reading frames (E6, E7, E1, E2, L1, and L2) and the spliced E1^E4 and E8^E2 mRNAs. This suggested that we recovered the genomes of two papillomaviruses associated with Mexican free-tailed bats (*Tadarida brasiliensis*). The current papillomavirus taxonomy is based on sequence identity across the L1 open reading frame (4). If two viruses share more than 60%, they fall into the same genus. Species within a genus collect viral “types” that share 70% to 90% sequence identity. A new papillomavirus type shares less than 90% sequence identity with other viruses (4, 71, 72). Both identified viruses share less than 90% identity with their closest relatives (Fig. 1). In consultation with the international animal papillomavirus reference center (73), we named these two novel papillomaviruses TbraPV2 (GenBank number MW922427) and TbraPV3 (GenBank number MW922428), respectively. TbraPV2 is 8,093 bp long, while TbraPV3 is 8,037 bp long. Based on pairwise sequence identity in the L1 open reading frame, both viruses are most closely related to TbraPV1 (Fig. 1). TbraPV2 shares 81.7% sequence identity with TbraPV1 and likely represents a new species in this unclassified genus. TbraPV3 shares 60.4% identity with TbraPV1, placing it in the same genus. However, the phylogenetic tree shown in Fig. 1 places TbraPV3 as an outgroup to a clade that contains HPV41, EdPV1, TbraPV1, and TbraPV2.

### Chiropteran PVs cospeciated with their hosts.

It has been demonstrated that cospeciation between PVs and their hosts is a major contributor to the papillomaviruses’ evolutionary history (1, 74, 75). In support of this theory, these novel bat papillomaviruses cluster with other previously described chiropteran papillomaviruses (Fig. 1). However, as for other papillomavirus-host relationships, bat-associated viruses are paraphyletic, suggesting that other evolutionary mechanisms like intrahost divergence or niche adaptation likely contribute to the papillomavirus phylogenetic tree (1, 76). While TbraPV2 and TbraPV3 cluster with several other chiropteran viruses, the larger clade consists of viruses infecting a wide array of mammals (red arrow in Fig. 1). In addition to 6 species of Chiroptera, the subtree contains 16 host species classified in 5 mammalian orders.

**FIG 2:**
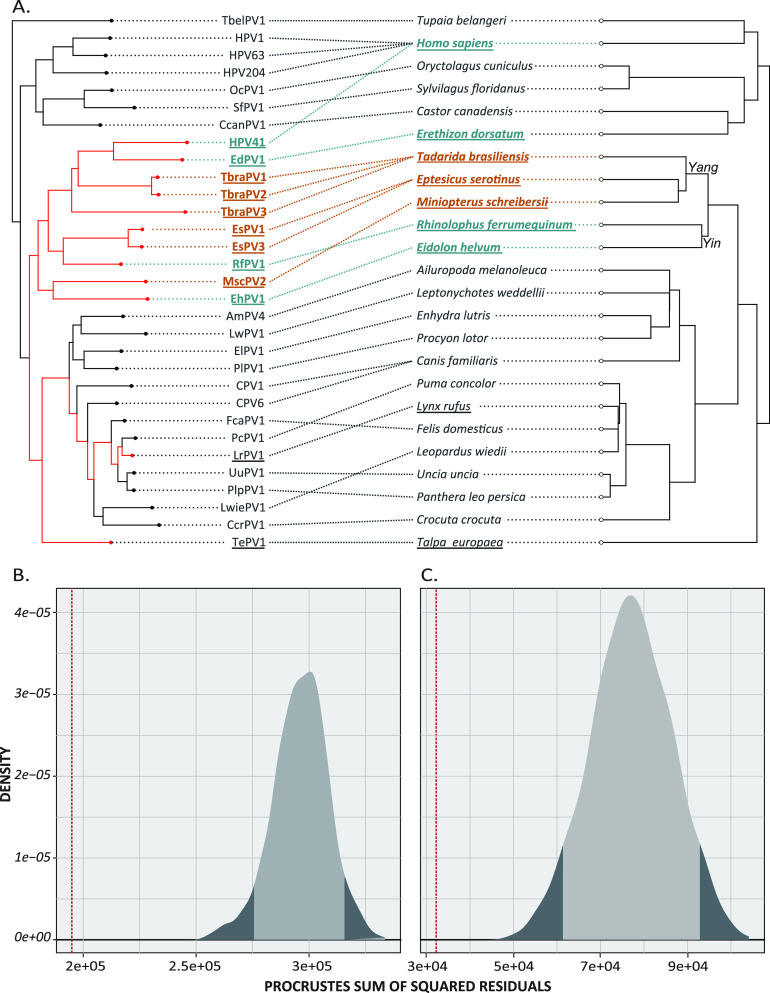
Coevolution of papillomaviruses. (A) Optimized tanglegram between subtree based on concatenated E1-E2-L1 maximum likelihood phylogenetic tree (Fig. 1) and associated host species. We downloaded the host species tree from www.timetree.org. Papillomaviruses are linked to their host phylogenies. Papillomaviruses associated with Chiroptera are highlighted: Yangochiroptera (orange) and Yinpterochiroptera (blue). The underlined virus/host pairs were used for the analysis in panel C. (B) Procrustean Approach to Cophylogeny analysis based on the interaction network and phylogenies shown in panel A supports the notion that papillomaviruses coevolved with their hosts. The observed best‐fit Procrustean superimposition (red dotted line) lies outside the 95% confidence interval (shaded area of the curves) of the distribution of network randomizations in the null model. (C) As in panel B but using a subset of the interaction network and phylogenies (underlined virus-host pairs in panel A).

We wanted to compare the evolutionary history of these diverse viruses to that of their hosts. Due to intrahost divergence and niche adaptation, papillomaviruses infecting the same host can be found in multiple phylogenetic tree clades. We extracted a subtree from the maximum likelihood (ML) tree to ensure that viruses with similar tissue tropisms and evolutionary histories were compared (Fig. 2) (77). This clade contains the newly identified TbraPV2 and TbraPV3 embedded within the largest monophyletic Chiroptera papillomavirus clade (red arrow in Fig. 1). We used a tanglegram to address our hypothesis of virus-host coevolution (Fig. 2A). This analysis rotates nodes in the host and virus phylogeny to optimize tip matching. Similarities between the host and virus phylogenetic relationships are indicated by parallel lines linking the virus to its host in their respective trees. Conversely, mismatches in the evolutionary history of the host and the virus show overlapping connecting lines. While there are some overlapping connections between papillomaviruses and their hosts, most virus-host pairs support the idea of cospeciation.

To quantify the degree of cospeciation, we focused on two data sets. First, we used the phylogenetic tree shown in Fig. 2A. Because it was previously demonstrated that members of the *Lambdapapillomavirus* genus coevolve with their hosts (78), we also tested a smaller subtree to avoid skewing the results (underlined in Fig. 2A). The host and virus phylogenetic trees were compared using the Wasserstein distance, estimated to be 0.205 and 0.284 for larger and smaller data sets. A Wasserstein distance of 0 indicates that the two trees are topologically identical, while a value of 1 indicates a complete lack of congruence between the trees (79). Therefore, the host tree predicts the virus tree, suggesting a role for cospeciation.

Finally, we used the Procrustean Approach to Cophylogeny (PACo). This approach evaluates congruency between distance matrices for each virus and associated host phylogenies (80, 81). The observed best‐fit Procrustean superimposition (1.08E5 and 3.22E4 for the larger and smaller data set, respectively; indicated by the red line in Fig. 2B and C) lies outside the 95% confidence interval of the ensemble of 1,000 network randomizations in the null model. Therefore, the data allow us to reject the null hypothesis that the papillomavirus host tree does not predict the virus tree and supports cospeciation as an important process for the evolution of this subclade of PVs and their hosts (80, 81).

### Yangochiropteran viruses have a reduced CpG content.

**FIG 3:**
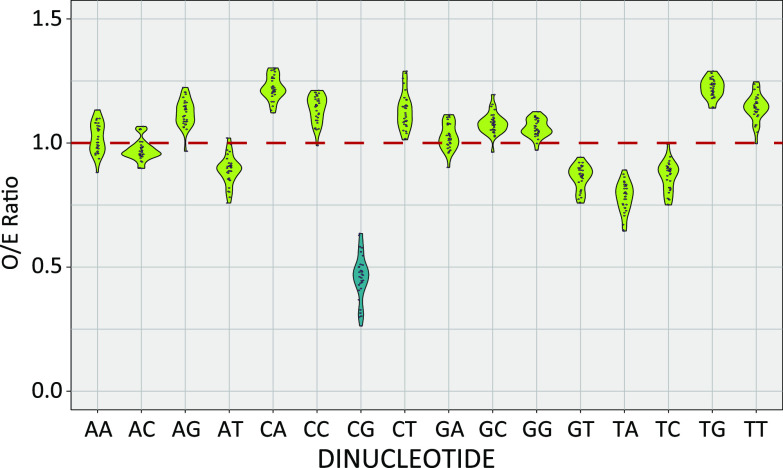
CpG dinucleotide sequences are significantly depleted in papillomavirus genomes. The observed-versus-expected (O/E) ratios of each dinucleotide in the papillomavirus genome sequences shown in Fig. 2 were calculated using a custom wrapper around the CompSeq program from the Emboss software suite. The red line at 1.0 indicates the ratio where a dinucleotide is seen as often as expected by chance.

We previously demonstrated that millions of years of virus-host cospeciation left historical evidence of this virus-host arms race in the papillomaviruses’ genomes. For example, the mucosal, cancer-causing viruses within the genus *Alphapapillomavirus* have a reduced TpC dinucleotide content, presumably due to evolutionary adaptations to APOBEC3 editing (33). To extend these studies, we calculated the observed/predicted ratio for each dinucleotide in the viruses shown in Fig. 2A. A ratio close to 1 indicates that a dinucleotide is seen in the sequence as often as expected based on each sequence’s nucleotide composition. Values lower than 1 suggest that a dinucleotide is depleted. While the ApC, ApT, GpT, TpA, and TpC ratios are significantly lower than 1 (one-sample *t* test *P*-value < 0.001), we observed the most significant decrease in the CpG dinucleotide ratio (Fig. 3). The median CpG content for these evolutionarily related viruses is 0.46. However, we noticed that the distribution has a long tail toward even lower values, suggesting that some viruses have a further-reduced CpG content (Fig. 3).

**FIG 4:**
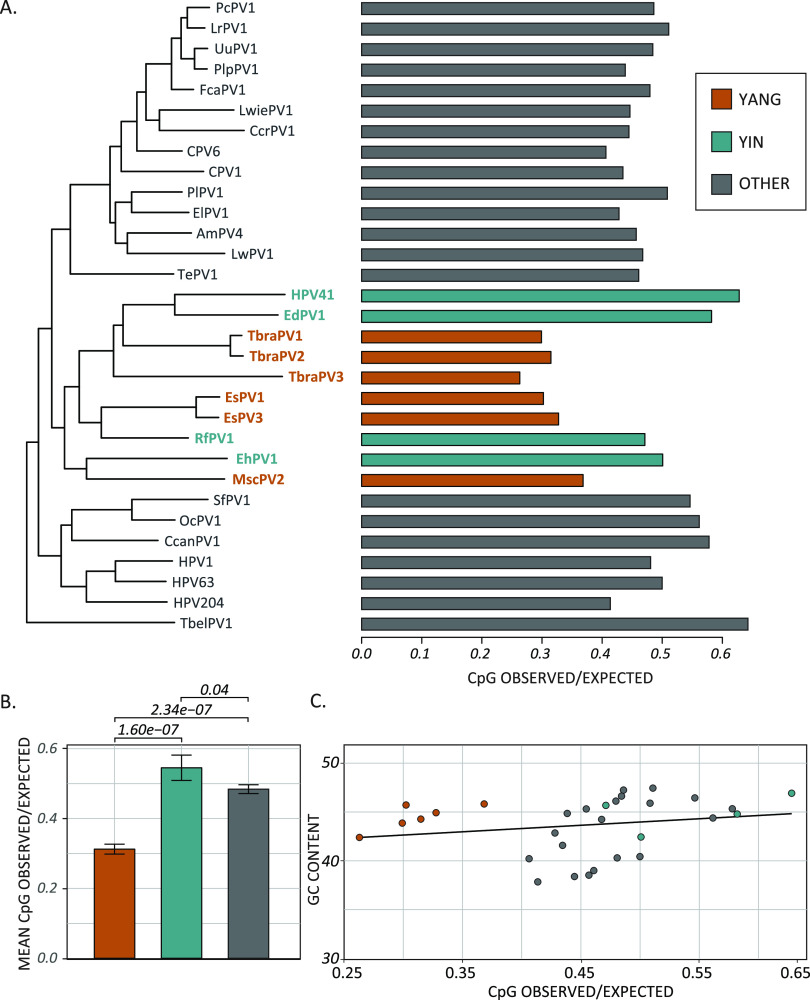
CpG content is significantly lower in papillomaviruses associated with Yangochiroptera than in related viruses. (A) A maximum likelihood phylogenetic tree is shown comparing the O/E ratios of CpG dinucleotides. Viruses infecting Yangochiroptera (red), Yinpterochiroptera (green), and related hosts (gray) are indicated. (B) Mean (±standard deviation) CpG O/E ratios for each group of viruses were compared using one-way ANOVA with Tukey’s *post hoc* test. (C) CpG O/E ratios are compared to total GC content for each viral genome in panel A.

When we plotted the CpG ratio for each virus on a phylogenetic tree (Fig. 4A), it became clear that the genomes of a subset of bat-associated papillomaviruses have a further-decreased CpG content. The order Chiroptera consists of two suborders, Yinpterochiroptera and Yangochiroptera (82–84). When we associated the viruses with their Yinpterochiroptera and Yangochiroptera hosts, the data demonstrate that the yangochiropteran papillomavirus genomes have even lower CpG values (orange bars in Fig. 4A) than the Yinpterochiroptera and other related papillomaviruses in the same phylogenetic clade (blue bars) and members of a closely related clade (gray bars) (analysis of variance [ANOVA] with *post hoc* Tukey test) (Fig. 4B).

This reduction in the CpG ratio could be due to an overall lower GC content. We compared the CpG ratio to total genomic GC content (Fig. 4C). This analysis demonstrated no correlation between the decreased CpG ratio and the total GC content (linear regression: *R*^2^ = 0.008 and *P* value = 0.27), and the reduced CpG ratio is not simply due to a lower GC content.

The viruses infecting bats in the Yangochiroptera suborder have a reduced CpG content, raising the possibility that the host species influences the CpG ratio and evolutionary trajectory of these viruses.

### CpG depletion alters codon usage without changing amino acid composition.

The CpG dinucleotide is present in 8 codons coding for five different amino acids. Therefore, reducing CpG dinucleotides is expected to lead to a bias in codon usage, amino acid composition, or both. Roughly 85% of the papillomavirus genome codes for viral proteins (2, 3). The viral genome contains several overlapping ORFs (1, 85). In some cases, this is a short overlap between the 3′ end of one ORF and the 5′ end of the downstream ORF (e.g., E6 and E7). In other cases, one ORF is wholly embedded within the coding region of another ORF (e.g., E4 embedded in E2 or E8 within E1). Since these overlapping regions are evolutionarily constrained at multiple codon positions (i.e., codon position 3 in frame 1 would be codon position 2 in the overlapping frame) (86), we removed these overlapping regions from the data (see Materials and Methods).

**FIG 5:**
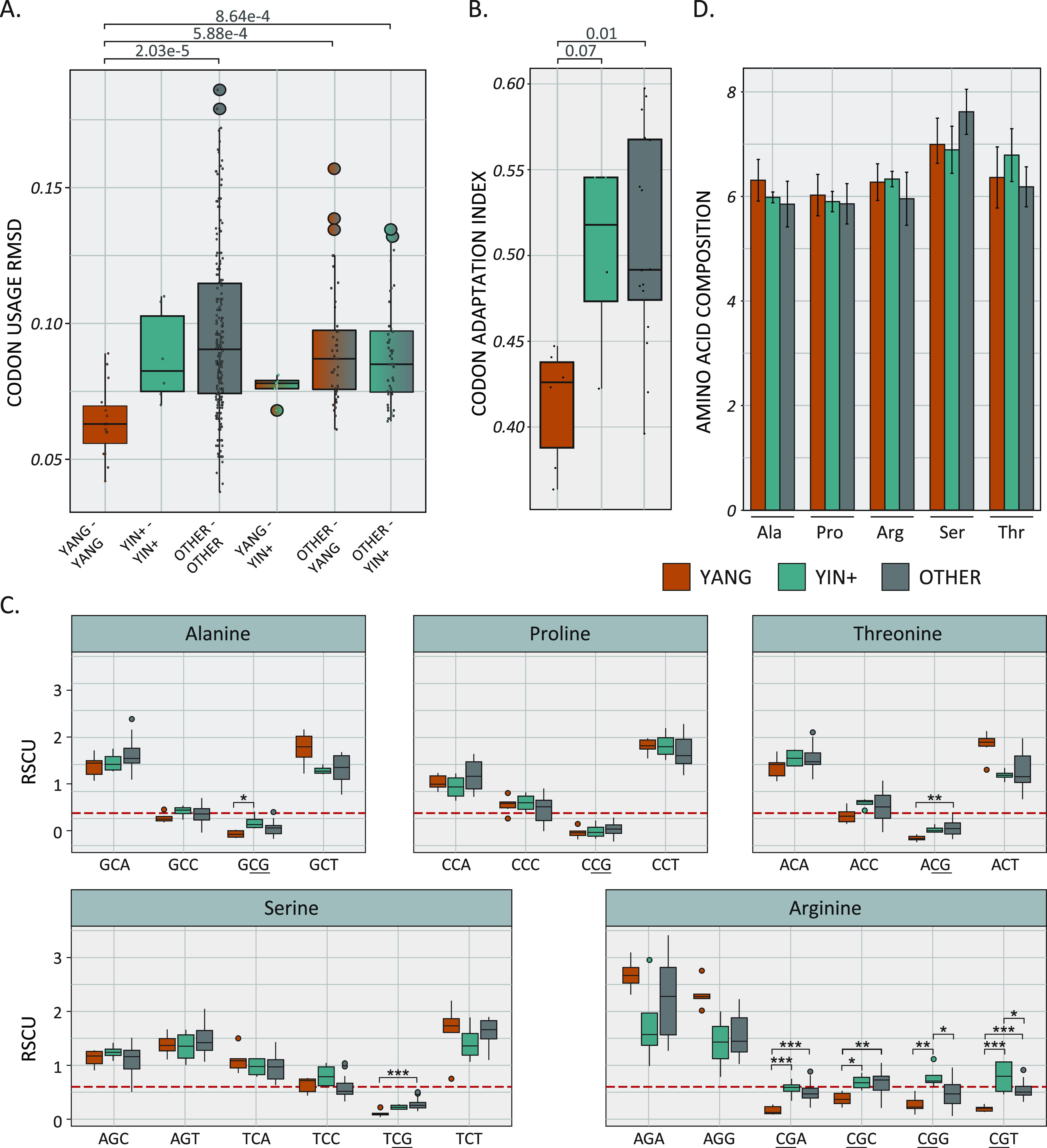
Yangochiroptera papillomaviruses have a restricted codon usage. (A) Codon usage tables for each virus in Fig. 2 were compared using the “codcmp” program from the Emboss software suite. Root-mean-square deviation (RMSD) values for each pairwise comparison are plotted as box-and-whisker plots with the outliers (colored circles) identified using Tukey’s method. Individual values are shown as single black dots. (B) The codon adaptation indices for a subset of viruses (see Materials and Methods) in Fig. 2 are plotted as box-and-whisker plots, with the outliers (colored circles) identified using Tukey’s method. Individual values are shown as single black dots. (C) RSCU values for the indicated amino acid/codons were calculated and plotted as box-and-whisker plots, with the outliers (colored circles) identified using Tukey’s method. RSCU values for each amino acid were compared using two-way ANOVA with Tukey’s *post hoc* test. *, *P* < 0.05; **, *P* < 0.01; ***, *P* < 0.001. (D) The amino acid composition was calculated as described in Materials and Methods. Mean values ± standard deviations are plotted.

We constructed codon usage tables for the nonoverlapping coding sequences for each of the papillomaviruses in the subtree described in Fig. 2A. These codon usage tables were compared using the Emboss “codcmp” tool to calculate codon usage differences. The more diverse the codon usage, the larger the differences between the tables. This analysis showed that the codon usages of Yangochiroptera papillomaviruses are more similar than those found when other viruses are compared (Fig. 5A). This suggests that the reduction in CpG leads to a more restricted availability of codons.

We used the codon adaptation index (CAI) to quantify the similarity between the synonymous codon usage of the nonoverlapping coding region for each virus and the synonymous codon frequency of a reference set of host genes. A CAI of 1 indicates that the virus uses the same codons as the host, while a virus that does not use any of the codons used by the host has a CAI of 0. It has been reported that the papillomaviruses’ codon usage is different from that of the host (87). Indeed, all tested virus-host pairs have a CAI below 0.6 (Fig. 5B). The Yangochiroptera papillomaviruses’ CAI is statistically reduced compared to that of the related viruses. These data support the notion that the Yangochiroptera viruses use different codons.

The relative synonymous codon usage (RSCU) value is the ratio of the observed frequency of one specific synonymous codon to the expected frequency (i.e., no codon usage bias). This ratio is an important measure of codon usage bias (88). RSCU values higher than 1.6 and lower than 0.6 indicate overrepresented and underrepresented codons (89). CpG-containing codons (underlined in Fig. 5C) are significantly underrepresented in this data set. For most amino acids, CpG-containing codons are further reduced in the Yangochiroptera papillomaviruses. In the case of arginine, the CpG-containing codons are statistically significantly depleted compared to the case with the related viruses (Fig. 5C). Since these codons’ CpGs are located in the 1st and 2nd positions of the codon, this depletion suggests that nonsilent mutations are evolutionarily preferred over maintaining a relatively high CpG content.

Of note, despite the biased codon usage, there is no change in the amino acid composition of the different viral proteins (Fig. 5D). Thus, despite reduced CpG content, the Yangochiroptera papillomaviruses likely coding for proteins with similar chemical properties.

### Natural selection in TLR9 of Yangochiroptera bats.

**FIG 6:**
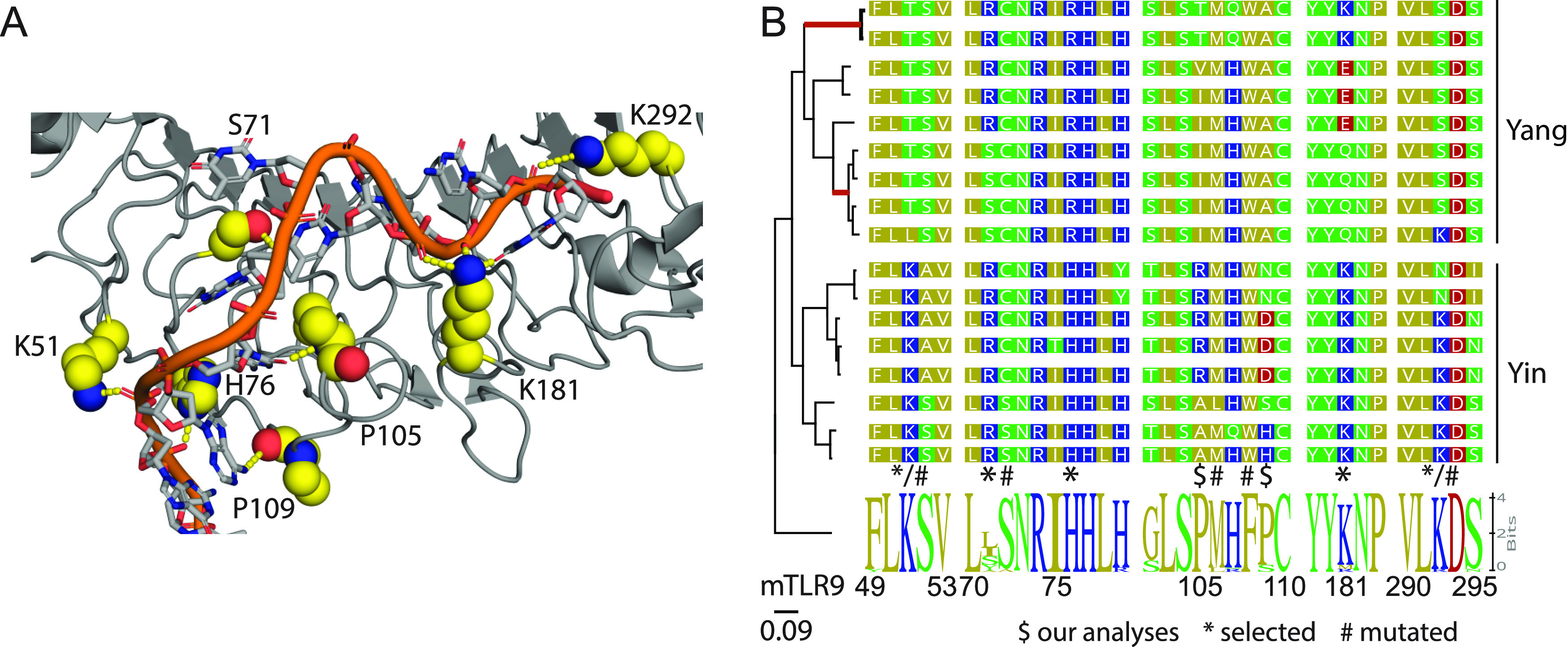
Yangochiroptera TLR9 is evolving under diversifying selection. (A) Structure of horse TLR9 in complex with agonistic DNA (PDB code 3WPC) (94). Amino acids of interest are highlighted. (B) Maximum likelihood phylogenetic tree of mammalian TLR9 sequences clusters Yangochiroptera and Yinpterochiroptera separately from the mammalian TLR9. Red branches display evidence of episodic diversifying selection as identified by aBSREL (92). Alignments show sequences of interest. The sequence logo is based on the alignment of 29 nonchiropteran TLR9 sequences. Numbering is based on the mouse TLR9. The analyses identified residues indicated by “$” as being selected using FEL. Residues indicated by “*” were previously identified as evolving under diversifying selection (66), while those indicated by “#” were identified as functionally important through site-directed mutagenesis (94).

Specific residues within the chiropteran TLR9 are under positive selective pressure (65, 66). We constructed a maximum likelihood phylogenetic tree to determine whether diversifying selection differentially altered Yangochiroptera and Yinpterochiroptera TLR9. As was previously reported, bat TLR9 sequences formed a monophyletic clade separate from other eutherian sequences (data not shown), not as a sister group to carnivores, ungulates, and cetaceans as is seen for other proteins (90). We used RELAX (91), adaptive branch site relative effect likelihood (aBSREL) (92), and fixed effect likelihood (FEL) tests (93) to detect evidence for evolutionary selection (see Materials and Methods). RELAX demonstrated that evolutionary selection intensity parameter (*k* = 6.07; likelihood ratio [LR] = 21.05) within the Yangochiroptera compared to the Yinpterochiroptera. Furthermore, aBSREL recovered evidence for episodic diversifying selection on the branches leading to the Yangochiroptera (Fig. 6B). Finally, FEL identified 7 sites under diversifying selection within the Yangochiroptera.

We mapped a subset of these residues as well as residues previously identified to be under diversifying selection (66) and sites shown to be functionally important for target recognition (94) onto the structure of TLR9 bound to target DNA (Fig. 6A). Many sites are highly variable compared to the mammalian consensus (Fig. 6B). Notably, there are apparent differences between the TLR9 sequences of Yangochiroptera and Yinpterochiroptera, specifically within the DNA recognition motif. *In silico* mutation analysis suggests that these Yangochiroptera-specific changes would alter how TLR9 recognizes its target DNA. Overall, these mutations lead to a reduction in the positive surface charge of TLR9. Within the Yangochiroptera, K51T leads to the loss of an ionic interaction with the phosphate backbone. The Arg at position 76 is much larger than the canonical His at this position, presumably impacting the interaction with the DNA. While the P105 residue van der Waals bonds with the C6 and T9 residues in the crystallized DNA, the I105 residue is too bulky to occupy the same conformation as the proline and would likely lead to a loss of the observed bend in the protein at this position. K181 is involved in ionic interactions with the DNA backbone. Q181 has no charge and is too short to interact with the DNA side chain. E181 would likely charge repel the DNA backbone. Finally, K292 interacts with the DNA backbone, but this interaction in absent in Yangochiroptera TLR9 due to the Ser residue at this position. Overall, the Yangochiroptera TLR9 DNA binding domain is predicted to be functionally different from the Yinpterochiroptera and the other mammalian TLR9 molecules.

### CpG depletion in a TLR9 recognition context.

**FIG 7:**
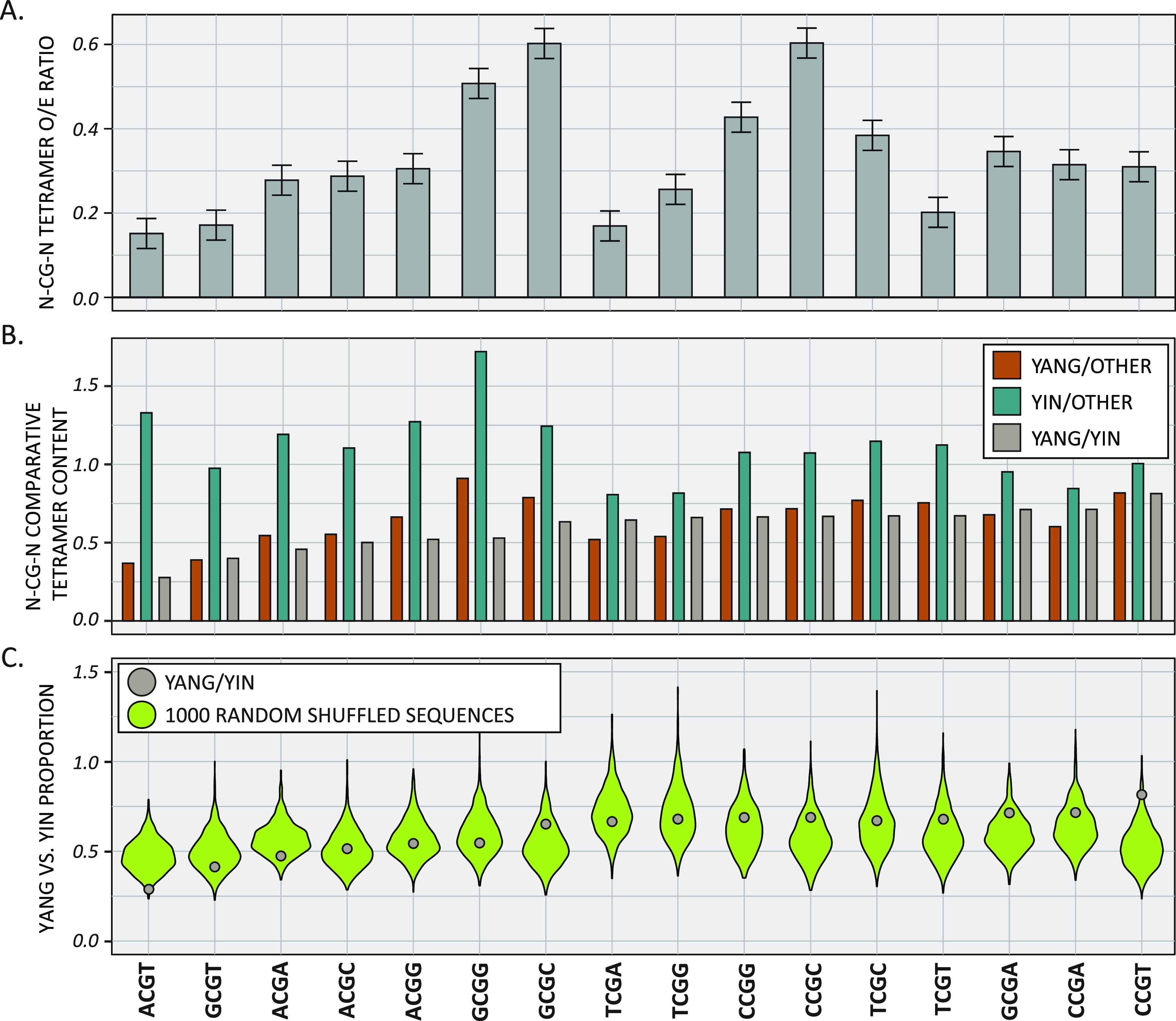
Yangochiroptera papillomaviruses depleted a TLR9 recognition motif from their genomes. (A) The O/E ratios of each nCGn tetramer in the Yangochiroptera papillomavirus genomes sequences were calculated using a custom wrapper around the CompSeq program from the Emboss software suite. Mean values ± standard deviations are plotted. (B) The O/E ratios of each nCGn tetramer in the different groups were calculated as for panel A. The proportion of these ratios provides a normalized view of tetramer depletion across papillomavirus genomes shown in Fig. 2. (C) The Yangochiroptera versus Yinpterochiroptera nCGn proportions (as in panel B) are plotted as brown dots and compared to 1,000 randomly shuffled sequences (green violin) plots. Only ACGT is statistically underrepresented in the Yangochiroptera.

Our data demonstrate that the Yangochiroptera TLR9 protein is under selective pressure, and papillomaviruses that infect these hosts have a decreased CpG content. We hypothesized that DNA recognition by TLR9 would lead to a loss of CpG in the context of a TLR9-specific PAMP. Thus, a specific set of tetramers should be depleted within viruses infecting Yangochiroptera bats. We calculated all tetramers’ observed/expected ratios and focused on those tetramers with a central CpG (nCGn) (Fig. 7A). This initial analysis indicated that ACGT, GCGT, TCGA, and TCGT are diminished in Yangochiroptera-infecting viruses. We calculated the average tetramer ratio for each group of viruses to normalize the differences in overall CpG content between Yangochiroptera and other viruses. We compared the proportions of Yangochiroptera-infecting viruses and Yinpterochiroptera-infecting viruses, Yangochiroptera-infecting viruses and other viruses, and Yinpterochiroptera-infecting viruses and other viruses (Fig. 7B) for each tetramer. For example, in Yangochiroptera papillomaviruses, ACGT was depleted 3- to 4-fold compared to other viruses or Yinpterochiroptera papillomaviruses, respectively. Conversely, this tetramer was not found to be depleted when Yinpterochiroptera-infecting viruses and other viruses were compared. Using a bootstrap method based on 1,000 randomly shuffled sequences (see Materials and Methods), the ACGT tetramer was identified as significantly depleted within Yangochiroptera-specific viruses (Fig. 7C). This tetramer is identical to the experimentally validated core mouse TLR9 recognition motif but is different from the human TLR9 PAMP (TCGw; w = T or A) (95–99). This suggests that papillomaviruses associated with Yangochiroptera specifically deplete CpG dinucleotides in the context of a known TLR9 PAMP.

### TLR9-mediated CpG depletion in human papillomaviruses.

To investigate whether TLR9 impacts the human papillomaviruses genome, we analyzed sequences of human oncogenic viruses and their close relatives within the genus *Alphapapillomavirus*.

**FIG 8:**
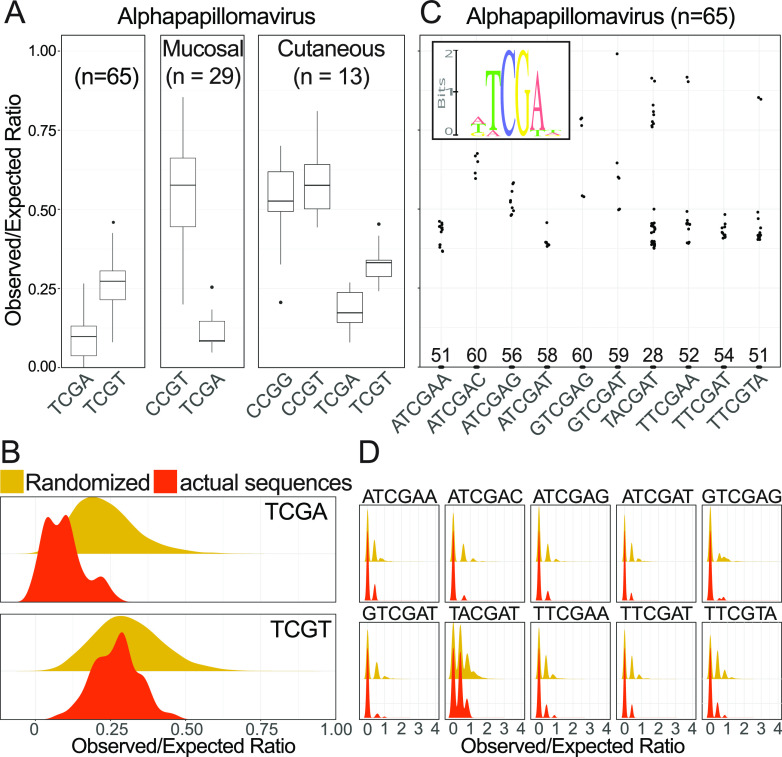
Human papillomaviruses depleted a TLR9 recognition motif from their genomes. (A) The O/E ratios for statistically underrepresented nCGn tetramers are plotted as box-and-whisker plots, with the outliers (circles) identified using Tukey’s method. All known HPVs within the *Alphapapillomavirus* genus are shown. We also separately analyzed the mucosal and cutaneous HPVs in this genus. (B) Statistical depletion of nCGn tetramers was based on comparison of distributions of the actual to randomized sequences. The distributions for TCGA and TCGT (*n* = 65 alphapapillomavirus genomes) are shown. (C) The O/E ratios for statistically underrepresented nnCGnn hexamers are plotted. Individual values are plotted as dots. Numbers near the *x* axis indicate the number of genomes in which the hexamer was not observed. (D) Statistical depletion of nnCGnn hexamers was based on comparison of distributions of the actual (red curves) to randomized (yellow curves) sequences. The distributions for the 10 hexamers identified in panel C (*n* = 65 alphapapillomavirus genomes) are shown.

Briefly, the observed/expected ratio for nCGn tetramers was calculated for all HPV genomes. To determine whether nCGn tetramers are reduced, we randomly shuffled the viral genomes 1,000 times and recalculated each tetramer’s observed/expected ratio. We compared the actual ratio distribution to the distribution based on these random sequences for each tetramer. Using this analysis, TCGA and TCGT were identified as underrepresented (Fig. 8A and B). This illustrates that CpG dinucleotides within a canonical human TCGw (w = T or A) context are significantly depleted.

We previously demonstrated a significant depletion of TpC within the genomes of alphapapillomaviruses related to evasion of APOBEC3 (33). Importantly, TpC depletion was more pronounced in mucosal HPV types than in cutaneous types (33). Therefore, if TpC depletion is the primary driver of the observed decrease in TCGw (Fig. 8A), we expect that cutaneous HPV genomes would not show TCGw reduction compared to mucosal HPV genomes. However, mucosal and cutaneous HPVs have similarly reduced TCGA contents (Welch’s *t* test, *P* > 0.01), suggesting an effect independent of APOBEC.

Similarly, out of 256 possible nnCGnn hexamers, 10 were found to be significantly depleted when we analyzed all the available human alphapapillomavirus sequences (Fig. 8C and D). The inset in Fig. 8C shows a sequence logo representation of these depleted sequences, highlighting the central TCGw motif. Importantly, TCGw is a canonical human TLR9 recognition sequence but is different from the sequence identified in the bat PVs. Together, these data suggest that TLR9 shapes the genomes of human papillomaviruses.

## DISCUSSION

The data presented here advance our understanding of papillomavirus evolution and host-pathogen interactions. Specifically, we provide evidence that papillomavirus genomes have evolved to avoid detection by TLR9. The implications of this finding for papillomavirus biology are discussed below.

### Papillomaviruses have a reduced CpG content in a TLR9-dependent manner.

We demonstrate that the genomes of viruses isolated from specific bat species have a highly reduced CpG content. A significant reduction of CpG sites in papillomavirus genomes has been previously documented (33, 56). However, the reason for this depletion is unclear. In mammalian genomes, CpGs are rare outside so-called CpG islands (100). This lack of CpG is believed to be because methylated CpGs are prone to deamination, resulting in C → T mutations, leading to a depletion of CpG sites in the mammalian genomes over evolutionary time. Our data in Fig. 3 show an increase in TpG and CpA. However, this increase does not appear to be of the same magnitude as the dramatic reduction in CpG seen in the same data set. This suggests that other factors must play a role.

The zinc finger antiviral protein (ZAP) acts as a broad-spectrum antiviral restriction protein that recognizes CpG-rich viral RNA, leading to RNA degradation and inhibition of translation (101). Interestingly, ZAP exploits host CpG suppression to identify non-self RNA. This may explain why multiple RNA viruses have a reduced CpG content (102, 103), independently from CpG methylation (104). ZAP was recently shown to restrict the replication of vaccinia virus Ankara (105) and human cytomegalovirus (HCMV) (106), demonstrating that ZAP recognizes CpG-rich viral RNA and can restrict CpG-rich DNA viruses. However, the consensus recognition site for murine ZAP was identified as CN_7_GNCG. In this motif, the CpG dinucleotide acts as the essential element, while the −2 G further enhances binding affinity 10-fold (107). Our tetramer analysis did not identify a downregulation of GNCG in Yangochiroptera-specific papillomaviruses (data not shown). Therefore, ZAP is unlikely to play a vital role during papillomavirus infection. However, this would need to be confirmed experimentally.

In contrast, we provide evidence that the depletion of CpG in papillomavirus genomes is partly due to the need to avoid detection by TLR9. Unmethylated CpG DNA motifs are recognized by TLR9, leading to an interferon and inflammatory cytokine-mediated antiviral response (50). By carefully analyzing the CpG contents of related chiropteran papillomaviruses, we demonstrated that viruses isolated from Yangochiroptera have a further-decreased CpG content. Importantly, we demonstrate that the Yangochiroptera TLR9 protein evolves under diversifying selection, specifically sites implicated in DNA recognition. Finally, by analyzing tetramer motifs, we showed that Yangochiroptera viruses are specifically depleted of ACGT, a known TLR9 recognition motif. Together, these data demonstrate that Yangochiroptera papillomaviruses deplete CpG, in the context of a TLR9 recognition motif, presumably in response to evolutionary changes within the TLR9 protein. This has important implications for papillomavirus biology and evolution. We demonstrated that human papillomaviruses show a similar reduction in CpG in the context of a human TLR9 recognition motif.

### Recognition of papillomavirus DNA in the endosomes during infectious entry.

Shortly after entry, papillomavirus virions are trafficked from early endosomes into acidic late endosome and multivesicular bodies, leading to capsid disassembly and uncoating of viral DNA (29). This uncoating may expose the viral DNA to TLR9, leading to an antiviral response. Importantly, TLR9 specifically recognizes unmethylated CpG motifs. Several studies have investigated the methylation state of oncogenic human papillomaviruses (108). While these studies have demonstrated that the viral DNA is methylated under specific conditions, it is unknown whether the packaged viral genome contains methylated CpG sites. However, we have some clues that would suggest that viral DNA inside the virion is likely hypomethylated. DNA methyltransferase 1 (DNMT1) is the primary cellular enzyme responsible for maintaining DNA methylation patterns after replication. The DNMT1 protein was found to be enriched in undifferentiated cells and is reduced as cells differentiate (109). Therefore, it is likely that the reduction in DNMT1 levels leads to a loss of methylation on the viral genomes destined for packaging and infection of the new tissue. Indeed, studies using HPV16-containing cells suggest that (parts of the) viral DNA is hypomethylated upon cellular differentiation (110).

A recent study showed that papillomavirus virions package DNA with histones enriched in modifications typically associated with “active” chromatin. Of interest, the authors demonstrate that the levels of H3K4me3 were enriched on virions compared to cellular controls. Conversely, virions were depleted in H3K9me3 (111). There is emerging evidence of active associations between histone lysine methylation and DNA methylation (112). For example, MeCP2 binds to methylated CpG (113) and recruits the Suv39h1/2 histone methyltransferases (114), increasing H3K9me marks (114, 115). In parallel, the H3K9me mark recruits DNMT3a/b to heterochromatin, leading to *de novo* methylation of CpG sites (116, 117). Since H3K9me3 is depleted in virions, it is tempting to conclude that virion DNA would be hypomethylated. Furthermore, H3K4me3 appears to be mutually exclusive with DNA methylation (118). H3K4me3 serves as a binding site for H3K9me2 demethylases (119), which would lead to loss of DNA methylation. Since virion DNA is enriched for H3K4me3, this further strengthens the hypothesis that viral DNA would be depleted in DNA methylation. Therefore, it seems reasonable to assume that the infecting genome would be hypomethylated and thus serve as a TLR9 PAMP.

As mentioned, recognition by TLR9 would lead to an antiviral response. Indeed, small interfering RNA (siRNA)-mediated knockdown of TLR9 has been shown to dramatically upregulate viral copy number and transcription following infection with HPV16 (120), suggesting that TLR9 can restrict HPV infection. The observation that despite a reduction in viral CpG, HPV16 infection is still improved by interfering with TLR9 (signaling) demonstrates an important rule in host-pathogen interactions. While the loss of all (unmethylated) CpG dinucleotides would avoid detection by TLR9, the virus can likely not wholly remove all CpGs from its genome. The virus and the host establish an uneasy balance.

### Direct evidence of coevolution between the virus and its hosts.

Coevolution alongside their hosts has been suggested to be an essential factor in the evolution of papillomaviruses (1, 78). However, the evolutionary history of PVs is complex. There has been no direct evidence in favor of coevolution between papillomaviruses and their hosts. The coevolution theory would predict that the virus would need to adapt when the host evolves a new antiviral skill. Therefore, as TLR9 evolves new functionalities, the virus would need to respond to preserve the balance between virus and host. Indeed, our data suggest that as Yangochiroptera TLR9 is undergoing diversifying selection, papillomavirus genomes infecting these bats further deplete their CpG content, specifically in the context of a known TLR9 PAMP. This is the first direct evidence of coevolution between this family of viruses and their hosts.

In the phylogenetic analysis, the viruses that infect Yinpterochiroptera and Yangochiroptera are not monophyletic but are present in three mixed clades (EsPV1, EsPV3, and RfPV1; MscP2 and EhPV1; and TbraPV1 to -3 [Fig. 2]). This suggests that these three main viral clades diverged before the ancestor of Yinpterochiroptera and Yangochiroptera split over 65 million years ago. As these ancestral viruses coevolved alongside the Yangochiroptera hosts, evolutionary pressures selected for loss of CpG. This occurred at least three separate times in the evolution of Yangochiroptera viruses. This strongly argues against a founder effect but favors recurring coevolutionary interactions.

### Immune evasion by nucleotide sequence editing.

We previously used computer modeling and reconstruction of ancestral alphapapillomavirus genomes to show that these viruses depleted TpC to allow for replication in tissues with high APOBEC3 expression, presumably to evade restriction by APOBEC3 by selecting for variants that contain reduced target sites in their genomes. We observed a similar correlation between TLR9 and CpG depletion, strengthening the notion that papillomaviruses avoid detection by the immune system by altering the nucleotide composition of their genomes without dramatically changing the protein-coding ability. This strategy likely allows the virus to maintain its core functionalities related to replication and transmission. Most viral proteins are multifunctional and interact with a plethora of host proteins. Nonsynonymous changes would likely disrupt these interactions.

### Oncogene-mediated reduction of TLR9.

We propose that HPVs evade detection by TLR9 in the endosome by depleting CpG dinucleotides from their genomes. Interestingly, the E6 and E7 oncoproteins of different human papillomaviruses have been shown to downregulate the expression of TLR9 (120–123). Importantly, E6 and E7 are not delivered to the cell during infection but require viral transcription after the viral genome is delivered to the nucleus and presumably has already been sensed in the endosome. This implies that the ability to degrade TLR9 may serve an additional function during the viral life cycle, independent of initial infection. This idea is supported by the observation that other viruses (Merkel cell polyomavirus, hepatitis B virus, and Epstein-Barr virus [EBV]) also interfere with TLR9 function during the maintenance phase of the infection (124–126). Nonetheless, this oncogene-mediated repression of TLR9 occurs after infection. Furthermore, it is not clear whether this phenotype is conserved in other (nonhuman) PVs. Indeed, our data demonstrating that human PVs also deplete CpG demonstrate that evading detection during infectious entry is critical.

### Conclusion.

In conclusion, phylogenetic and genomic analyses of novel bat-associated viruses TbraPV2 and TbraPV3 demonstrate that host-virus interaction, specifically evasion of the innate immune system, affects the evolution of papillomaviruses. The impact of TLR9 is also present on oncogenic HPVs. These data suggest that TLR9 acts as a restriction factor for papillomavirus infection. Furthermore, we provide the first direct evidence for coevolution between papillomaviruses and their hosts.

## MATERIALS AND METHODS

### Sampling and sample processing.

We captured bats in mist nets set over water sources, extracted them from the nets, and put them in brown paper bags. Bats were held in bags for 20 min, removed, measured, and weighed. Individuals were identified as species in the field using forearm length and weight metrics. Feces and urine were collected from the bag or swabbed directly off the bat using a PurFlock 0.14-in. Ultrafine swab (Puritan, Guilford, ME). All feces and urine samples were put into tubes containing 0.5 mL of buffer consisting of 1× phosphate-buffered saline (PBS) and 50% glycerol. These samples were held on ice until being returned to the lab and stored in a −80°C freezer. We followed all applicable international, national, and institutional guidelines for the care and use of animals during sampling. The study was approved by the University of Arizona Institutional Animal Care and Use Committee (permit 15-583). Permits from the Arizona Department of Game and Fish were numbered SP506475.

Of each of the fecal samples, 5 g was homogenized in SM buffer, and the homogenate was centrifuged at 6,000 × *g* for 10 min. The supernatant was sequentially filtered through 0.45-μm and 0.2-μm syringe filters, and viral particles in the filtrate were precipitated with 15% (wt/vol) polyethylene glycol 8000 (PEG 8000) with an overnight incubation at 4°C followed by centrifugation at 10,000 × *g* as described previously (69). The pellet was resuspended in 500 μL of SM buffer, and 200 μL of this was used for viral DNA extraction using the High Pure viral nucleic acid kit (Roche Diagnostics, Indianapolis, IN). The total DNA was amplified using rolling-circle amplification (RCA) with the TempliPhi 2000 kit (GE Healthcare, USA), and the RCA products used to prepare Illumina sequencing libraries were then sequenced at Novagene Co. Ltd. (Hong Kong) on an Illumina NovaSeq 6000. The paired-end raw reads were trimmed using default settings within Trimmomatic v0.39 (127). The trimmed reads were *de novo* assembled using k-mer values of 33, 66, and 77 within metaSPAdes v3.12.0 (128). The resulting contigs greater than 500 nucleotides were analyzed by BLASTx (129) against a local viral protein database constructed from available NCBI RefSeq viral protein sequences (https://ftp.ncbi.nlm.nih.gov/refseq/release/viral/).

### Calculation of nucleotide frequencies.

We used a custom python script to determine a single observed-versus-expected (O/E) dinucleotide ratio across the entire viral genome, leveraging the CompSeq program from Emboss (33). We estimated the expected frequencies of dinucleotide “words” based on the observed frequency of single bases in the sequences. We analyzed only the forward frame.

The tetramer and hexamer contents for each genome were calculated as described for the dinucleotides.

To normalize tetramer content across groups of viruses, we calculated the average O/E ratio across the different groups. These average O/E ratios were compared as indicated in the figure legends. To test whether any of the tested tetramer depletions were statistically significant, we randomly shuffled each viral genome. To ensure that each randomly shuffled sequence would maintain the same dinucleotide ratio as the original sequence, we used the Altschul and Erickson algorithm (130) as implemented by Clote and colleagues (131). Based on these shuffled sequences, we calculated the above Yangochiroptera/Yinpterochiroptera ratio. This was repeated 1,000 times to establish a null distribution. The 1st percentile was used as a significance cutoff.

Complete genomes for human papillomaviruses belonging to the genus *Alphapapillomavirus* (*n* = 65) were downloaded from the PV database (PaVE), and tetramer and hexamer O/E ratios were calculated using the CompSeq program from Emboss. For statistical analyses, we randomly shuffled each viral genome 1,000 times as described above. We used a Wilcoxon rank sum test to compare the actual alphapapillomavirus genomes to the shuffled genomes (*P* < 0.01). Mucosal and cutaneous subsets were identified as described previously (33, 71) and analyzed further.

### Phylogenetic analyses.

Annotated sequences (*n* = 409) were downloaded from the PaVE genome database. A maximum likelihood (ML) phylogenetic tree was constructed as described previously (132). The amino acid sequences for E1, E2, and L1 of all known papillomaviruses and the new TbraPV2 and TbraPV3 were individually aligned in MAFFT v7.3 (133, 134) using the L-INS-I algorithm. PartitionFInder2 was used to determine a partitioning scheme for the concatenated E1-E2-L1 alignment under the corrected Akaike information criterion (AICc) (135). This process separately identified each gene to evolve under the LG+I+G+F evolutionary substitution model. The concatenated E1-E2-L1 alignment was used to infer the best ML phylogenetic tree using RAxML-HPC v8 (136) on CIPRES Science Gateway (137) followed by a rapid bootstopping analysis. *A posteriori* bootstopping was automatically rendered in RAxML under the extended majority-rule consensus tree criterion (autoMRE). The best ML tree was rendered and edited in RStudio using the “ggtree” (138) and “treeio” (139) packages.

Taxonomic classification of TbraPV2 and TbraPV3 was based on pairwise sequence identity. The L1 sequence of each pair was aligned at the amino acid level using the L-INS-I algorithm as implemented within MAFFT v7.3 (133, 134). This way, the alignments preserve the codons. The resulting alignments are back-translated to nucleotide alignments and used to calculate pairwise sequence identity.

### Coevolution analysis.

We used functions in the R “ape” (140) package to extract a well-supported clade from the maximum likelihood phylogenetic tree. The extracted clade represents viral sequences in the genera *Lambdapapillomavirus*, *Mupapillomavirus*, *Nupapillomavirus*, *Kappapapillomavirus*, *Sigmapapillomavirus*, and *Dyosigmapapillomavirus* and the most extensive set of known bat papillomaviruses, including the two novel bat papillomaviruses described in this paper. We downloaded a corresponding host species phylogeny from TimeTree (www.timetree.org) (141–143).

A tanglegram representing the evolutionary relationship between the papillomaviruses and their hosts was constructed in the “phytools” package (144). Phytools optimizes tanglegrams by rotating nodes in the rooted phylogenies to minimize crossings between connecting lines between the trees.

An additional subtree was extracted to minimize the impact of the genus *Lambdapapillomavirus*. The viral types included in this smaller data set are underlined in Fig. 2. To assess the congruency between PV and host phylogenies, we used the Procrustes Approach to Cophylogenetic Analysis (PACo) (80) as implemented in R for both data sets. PACo uses cophenetic distance matrices for the virus and host trees and an association matrix of virus-host interactions. A Procrustean superimposition of the sum of squared residuals was generated from 1,000 network randomizations under the “r2” randomization model to assess statistical significance. Under this model, host specialization is assumed to drive virus diversification (81). The values for the actual tree comparisons were considered statistically significant if they fell outside the 95% confidence interval.

To quantify the similarity between the virus and host phylogenies, we calculated the Wasserstein distance using the “castor” R package (145). The Wasserstein distance is based on a modified graph Laplacian (MGL). The MGL uses evolutionary distances between nodes to construct a matrix that maintains branch length and tree topology information and allows for comparing phylogenies from different species. Specifically, the differences between a phylogeny’s degree matrix (sum of branch lengths from one node *n* to all others) and distance matrix (sum of all pairwise branch lengths) are calculated to generate a spectrum of eigenvalues. To calculate a normalized MGL (nMGL), the MGL is divided by the degree matrix. The normalized MGL is useful when comparing trees on different timescales by emphasizing topology oversize (79). The Wasserstein distance represents the largest eigenvalue from the spectra of the modified graph Laplacians. All eigenvalues from the graph Laplacian spectrum were used to calculate the Wasserstein tree distance. The Wasserstein tree distance metric calculated in “castor” considers branch length and tree topology and takes values between 0 and 1. Identical tree topologies would have a Wasserstein distance of 0.

### Analysis of codon usage.

We used a custom script to delete all overlaps between open reading frames. Briefly, overlaps between E6 and E7, E1 and E8, E2 and E4, and L2 and L1 were removed when present. For each overlap, entire codons were removed as not to introduce frameshifts. These sequences were concatenated and further analyzed.

We used CUSP (Emboss suite of tools) to generate codon usage tables for each virus. These tables were compared using codcmp (Emboss suite of tools). For each codon in the table, codcmp calculates the proportion of a codon to the total number of the codons in the table. Next, codcmp calculates the difference between the usage fractions in both tables. We plotted the root mean squared distance (RMSD) between pairs of tables.

RSCU is a simple measure of nonuniform usage of synonymous codons in a coding sequence (88). We used the R package SeqinR (146) to calculate the RSCU values.

Codon adaptation indices (CAIs) were calculated using the Python CAI package (147). The CAI compares viral codon usage to that of the host. Host codon usage was based on the ACTB, glyceraldehyde-3-phosphate dehydrogenase (GAPDH), HPRT1, TUBB, EGFR, KRT5, KRT10, and IFN-κ genes. Coding sequences for these genes were downloaded from GenBank when available. For Erethizon dorsatum (BioProject PRJNA523436), *Tadarida brasiliensis* (BioProject PRJNA399430), and Eidolon helvum (BioProject PRJNA209406), we BLAST searched the closest identified orthologs against the scaffold genome for each species. In some cases, manual curation was necessary to ensure that putative genes were in frame. Accession numbers and final sequences for these genes are provided at https://github.com/KVDlab/King-2021.

The amino acid composition for each viral sequence was calculated as described previously (148).

### Diversifying selection of analysis of Yangochiroptera TLR9.

TLR9 sequences were downloaded from NCBI and translated into putative proteins. The amino acid sequences were aligned using MAFFT v7.3 and back-translated into codon-aware nucleotide alignments. FastTree (149) was used to construct a maximum likelihood phylogenetic tree using the GTR substitution model of evolution.

To determine whether the strength of natural selection intensified along the Yangochiroptera compared to the Yinpterochiroptera, we used RELAX. After fitting a codon model with three ω classes to the phylogeny (null model), RELAX then tests for changes to the selection intensity by introducing selection parameter *k*. The null and alternative models are compared using a likelihood ratio test. A significant result (*k* > 1) indicates that selection strength has been intensified along the test branches (91).

Aadaptive branch site random effects likelihood (aBSREL) was used to test if positive selection has occurred on the branches leading to Yangochiroptera. aBSREL determines whether a proportion of sites have evolved under positive selection (92).

Finally, we used fixed effects likelihood (FEL) to infer nonsynonymous (dN) and synonymous (dS) substitution rates on a per-site basis. This method assumes that the selection pressure for each site is constant along the entire phylogeny. FEL fits an MG94xREV model to each codon site to infer nonsynonymous and synonymous substitution rates at each site. A likelihood ratio test determines if dN is significantly greater than dS.

### Statistical analysis.

One- or two-way analysis of variance (ANOVA) was used where appropriate. Data are presented as box-and-whisker plots, with Tukey’s method for outliers noted as distinct data points. All graphs were generated using R. Results were considered statistically significant at a *P* value of <0.01.

### Data availability.

We retrieved full-length reference sequences from PaVE (https://pave.niaid.nih.gov/). Data and code for all analyses are available from https://github.com/KVDlab/King-2021. TbraPV2 (MW922427) and TbraPV3 (MW922428) sequences are available in GenBank. Raw sequencing data are available in SRA (PRJNA718335).
